# Mobility and coalescence of stacking fault tetrahedra in Cu

**DOI:** 10.1038/srep09084

**Published:** 2015-03-13

**Authors:** Enrique Martínez, Blas P. Uberuaga

**Affiliations:** 1 Material Science and Technology Division, MST-8, Los Alamos National Laboratory, Los Alamos, 87545 NM, USA

## Abstract

Stacking fault tetrahedra (SFTs) are ubiquitous defects in face-centered cubic metals. They are produced during cold work plastic deformation, quenching experiments or under irradiation. From a dislocation point of view, the SFTs are comprised of a set of stair-rod dislocations at the (110) edges of a tetrahedron bounding triangular stacking faults. These defects are extremely stable, increasing their energetic stability as they grow in size. At the sizes visible within transmission electron microscope they appear nearly immobile. Contrary to common belief, we show in this report, using a combination of molecular dynamics and temperature accelerated dynamics, how small SFTs can diffuse by temporarily disrupting their structure through activated thermal events. More over, we demonstrate that the diffusivity of defective SFTs is several orders of magnitude higher than perfect SFTs, and can be even higher than isolated vacancies. Finally, we show how SFTs can coalesce, forming a larger defect in what is a new mechanism for the growth of these omnipresent defects.

The formation of stacking fault tetrahedra (SFTs) is a primary problem in structural face-centered cubic (fcc) metals as their presence dramatically modifies the mechanical properties of the material^1^,^2^. SFTs present an obstacle for dislocation motion, which causes hardening, embrittlement and plastic instabilities^3^,^4^,^5^,^6^,^7^,^8^,^9^,^10^. SFTs might be produced by heavy plastic deformation^11^ or quenching from temperatures close to the melting point^12^,^13^. In materials under irradiation, used for either medical, space or energy generation applications, SFTs are also ubiquitous, as irradiation-created vacancies agglomerate and collapse to create more stable SFT defects^14^,^15^,^16^,^17^,^18^. Experimentally it is observed that at temperatures below about 473 K a fine distribution of small SFTs (2.5 nm) forms in Cu samples irradiated with neutrons up to doses on the order of ~1 dpa. In contrast, above 500 K, faceted voids start to appear with larger sizes^19^,^20^,^21^. Recent studies have proposed several mechanisms for the growth of medium-to-large size SFTs^22^,^23^, namely, vacancy aggregation and expanding ledges. Because the edges of SFTs are formed by stair-rod dislocations and they are predicted to be sessile^24^ as their glide plane is not a compact {111} plane (actually W. T. Read called them *supersessile* dislocations), the SFT is commonly thought to be an immobile defect. Were this true, how do large voids form at elevated temperatures and why are small SFTs not observed in this temperature regime? Even for the smallest of vacancy clusters, containing as few as six vacancies, molecular dynamic (MD) simulations predict that the SFT structure is the most stable configuration in this temperature range. Thus, with the exception of vacancy clusters containing less than six vacancies^25^, large vacancy clusters which universally adopt the SFT structure should be essentially immobile. We show in this report that this statement does not hold true and conventional wisdom happens to be incorrect on this matter.

In the following we have performed MD and temperature accelerated dynamics (TAD)^26^ simulations (see the Methods section) of SFTs in Cu to demonstrate that, contrary to the common expectation, SFTs show large mobilities even for sizes of ~1.2 nm. We find that they can escape their minimum energy configuration with relatively small barriers, forming distorted SFT structures, and, as a consequence, their center of mass (CoM) will follow a random walk and migrate long distances in relatively short time scales. Moreover, we show that for defective SFTs, *i.e.* SFTs which are not formed by a *magic* number of vacancies (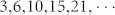), their mobility is several orders of magnitude higher than for the magic cases, and strikingly, even higher than isolated single vacancies. This high mobility leads to the possibility of interactions between multiple SFTs and we show how a 15-vacancy (V) SFT reacts with a 13-V SFT to form a 28-V perfect SFT, which is a novel growth mechanism for SFTs that was not previously described in the literature. The high mobility for defective SFTs is a plausible explanation of the experimentally observed low density of SFTs at high temperatures since they could diffuse and coalesce to form the reported large faceted voids.

## Results and Discussion

**Figure 1:**
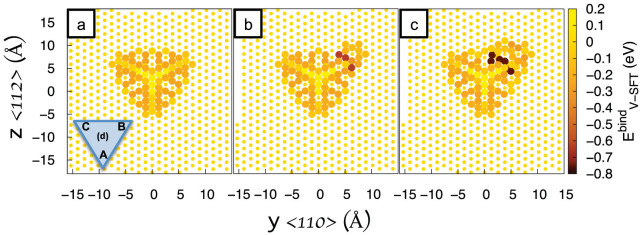
Vacancy-SFT binding energy for (a) a perfect 15-V SFT; (b) a defective 16-V SFT and; (c) a defective 17-V SFT. The triangular inset in (a) represents the sample orientation in Thompson notation.

As a growth mechanism, we have first studied the interaction energy between a single vacancy and a 15-V SFT in a sample with 3441 atoms (see Fig. 1). The binding energy of a vacancy to the SFT has been defined as
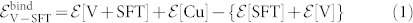
where 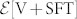 is the energy of the system with the vacancy and the SFT, 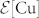 is the energy of the pure Cu system, 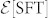 is the energy of the system with the SFT only and 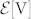 is the energy of the Cu system with one vacancy. We find that there are sites on the surface of the SFT which strongly attract vacancies 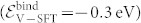. Calculations for a 16-V SFT and 17-V SFT have also been carried out (Fig. 1(b) and (c)) with binding energies as high as 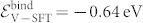 and 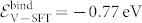 respectively. Therefore, there is a significant thermodynamic driving force for vacancies to agglomerate at these sites generating defective SFTs.

**Figure 2:**
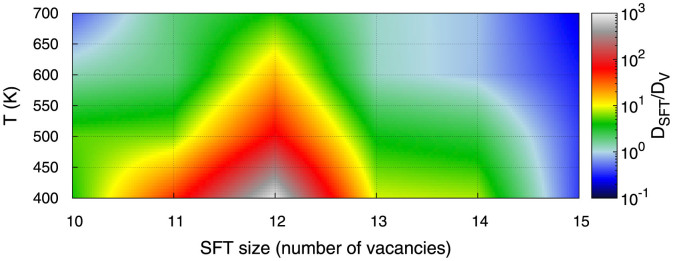
Map of the diffusion coefficient of a SFT (D_SFT_) relative to the mono-vacancy diffusion coefficient (D_V_) as a function of its size and temperature, extracted from MD simulations. Temperatures range from 400 to 700 K.

These defective SFTs are surprisingly mobile. We have performed MD simulations for SFTs ranging in size from 10 to 15 vacancies for 600 ns and extracted the diffusion coefficient of the SFT CoM. This has been done for temperatures between 400 K and 700 K. Figure 2 shows the SFT diffusion coefficient relative to the diffusion coefficient of an isolated vacancy. We find that the diffusivity is not monotonic with the SFT size. In fact, there is a maximum when the number of vacancies is 12, for what might be considered the most defective SFT structure among those studied in this range. We also observe that the mobility for perfect SFTs (10 and 15 vacancies) decreases with size; the 10-V SFT has a higher mobility than the 15-V SFT. Further, the diffusivity changes by about four orders of magnitude at low temperatures from the maximum mobility of the defective 12-V SFT to either of the perfect SFTs (10 or 15 vacancies). However, the most striking result might be the fact that the mobility of a defective SFT can be up to three orders of magnitude larger than that of a single vacancy at low temperatures.

**Figure 3:**
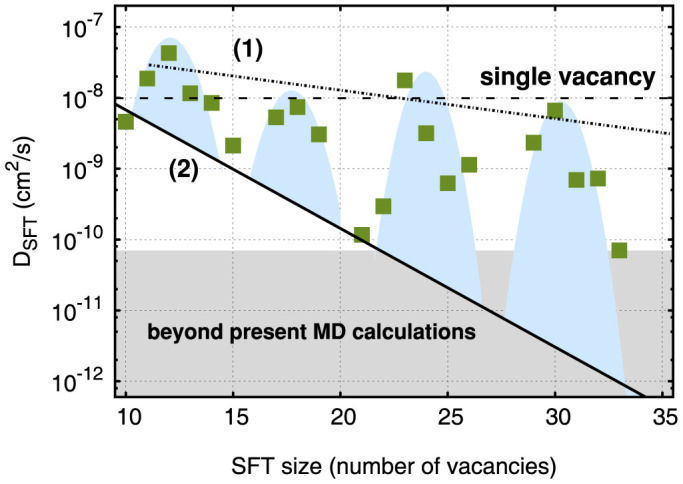
Diffusion coefficient at 700 K of several SFT configuration depending on the number of vacancies composing the defect. The green dots show the MD results and the light blue shade is a fit to the data. The maximum diffusivity of defective SFTs (1) and the minimum diffusivity of perfect SFTs (2) have also been fitted. The diffusivity of a single vacancy is also shown for comparison.

These trends extend to even larger sized SFTs. We have determined the diffusivities of selected larger SFTs containing up to 33 vacancies at one particular temperature of 700 K (see Fig. 3). Consistent with the observation noted above for the 10 and 15-V SFTs, we observe that the mobility of perfect SFTs decreases exponentially fast with the SFT size. In fact, perfect SFTs larger than 21 vacancies were not observed to move during the MD simulations. The exponential function that best fits the perfect SFT diffusivities was 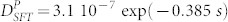, where *s* is the number of vacancies in the SFT. On the other hand, the diffusivity of defective SFTs decays substantially slower than for the perfect defects. The best exponential fit to the simulation results for the fastest SFTs in each size range gives 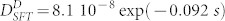. For comparison we also plot the diffusion coefficient of a single vacancy (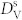) calculated by the same methodology as for the SFTs. We obtain a value of 
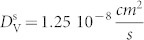
 which is in good agreement with the analytical diffusion coefficient of a vacancy in bulk Cu, 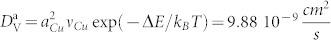. In this last expression 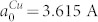 is the Cu lattice parameter, *ν_Cu_* = 6.794 10^11^ *s*^−1^ is the vacancy prefactor^27^ as calculated with Mishin's potential, Δ*E* = 0.688 eV is the vacancy migration barrier as calculated via the nudged elastic band method^28^,^29^,^30^,^31^, *k_B_* is the Boltzmann constant and *T* = 700 K is the temperature. Remarkably, we see that the diffusivities for defective SFTs are comparable to the diffusivity of a single vacancy for the analyzed sizes. Indeed, small SFTs, containing as many as 11–13 vacancies, exhibit mobilities higher than that of the mono-vacancy. These results align with the ones obtained by Sabochick *et al.*^25^ for vacancy clusters containing up to four vacancies.

**Figure 4:**
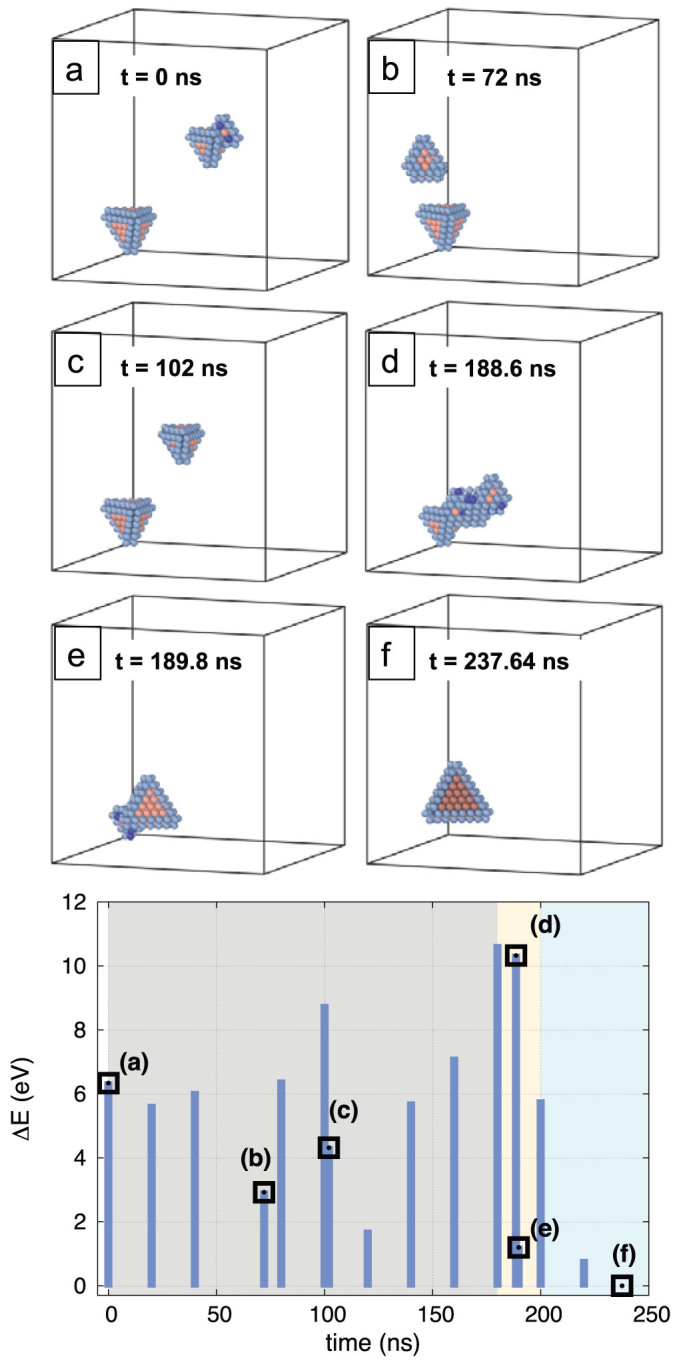
Reaction between a 15-V SFT and a 13-V SFT. (a) Initial configuration; (b) and (c) the 13-V SFT migrates randomly in the system until it encounters the 15-V SFT; (d) SFTs react forming an unstructured complex; (e) the complex rearranges itself to form a defective SFT; (f) the defective SFT finds the minimum energy configuration forming a perfect 28-V SFT. The bottom plot shows the evolution of the energy relative to the final configuration (blue bars) with black squares referring structures (a) to (f). In the gray area the SFTs diffuse randomly, in the yellow region the reaction takes place and in the blue zone the perfect 28-V SFT is formed.

The fact that defective SFTs have mobilities comparable to that of the mono-vacancy, means interactions between SFTs are probable. We have studied one such reaction, between a defective 13-V SFT and a perfect 15-V SFT (see Supplementary Material for a movie of the interaction process). Figure 4 shows the dynamics of this process at 700 K. The mobility of the perfect 15-V SFT is negligible on these time scales but the 13-V SFT performs a random walk in the sample until it encounters the larger SFT. At that point, a disordered structure replaces both SFTs (Fig. 4 (d)). After about 1.1 ns this structure transforms into a defective SFT (Fig. 4(e)), which follows a collective rearrangement to finally reach the minimum energy configuration in which a perfect SFT with 28 vacancies is formed (Fig. 4(f)).

To provide further insight into the mechanisms responsible for these high mobilities, we performed TAD simulations of SFTs containing 9, 10, and 11 vacancies (the 10-V SFT is perfect). The details of the TAD algorithm can be found in the Methods section and Ref. 26.

**Figure 5:**
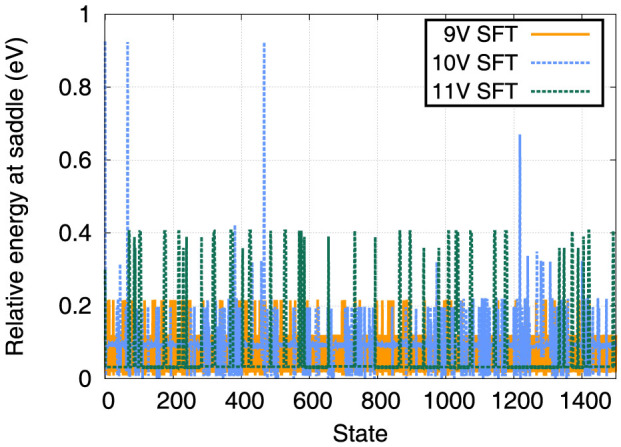
Saddles visited during TAD simulations of 9, 10 and 11-V SFTs. The energies of the saddles are measured in eV relative to the minimum in which the system resided when the saddle was visited. The large saddle at state number 468 for the 10-V SFT corresponds to the deformation of newly reformed perfect SFT just prior.

Figure 5 shows the magnitude of barriers executed during the TAD simulations where the height of the barriers is measured relative to the minimum the system was visiting when the event was executed. The first 1500 saddles accepted in the TAD simulations are shown. First, if one compares the saddles visited during the simulation of the 9-V SFT and the 11-V SFT, the sizes of barriers are qualitatively different. These two SFTs are both defective, but compared to the perfect 10-V SFT, the 9-V SFT can be considered as a 10-V SFT plus a self-interstitial, while the 11-V SFT is a 10-V SFT plus a vacancy. This qualitative difference is reflected in the different spectra of barriers visited in the two cases. In the case of the 9-V SFT, the barriers are typically smaller, with three distinct sets of barriers at about 0.01 eV, 0.12 eV, and 0.22 eV. In contrast, for the 11-V SFT, there are only two distinct sets of barriers, one at 0.03 eV and another at 0.39 eV. The 9-V SFT, which has interstitial character, has much smaller barriers for rearrangement, corresponding to a “surface interstitial” moving along the stair-rods of the SFT. In contrast, for the 11-V SFT, the “surface vacancy” has significantly higher barriers.

Interestingly, the perfect 10-V SFT, once it has deformed, exhibits a combination of the two types of events. There are both low barrier events of about 0.01 and 0.2 eV, representative of interstitial character, and 0.3 eV barrier events representative of vacancy character. These types of events are only active once the perfect 10-V SFT has distorted, a process which requires a barrier of 0.93 eV. This large barrier holds the key to the low mobility of the *magic* SFTs seen in MD. Before they can diffuse, SFTs must first deform, creating surface defects of both interstitial and vacancy character. Further, the barrier to nucleate these surface defects is a function of the SFT size. For 10-V SFTs, the barrier is 0.93 eV, but this barrier increases to 1.6 eV for 15-V SFTs and 1.6–1.9 eV for 21-V SFTs.

**Figure 6:**
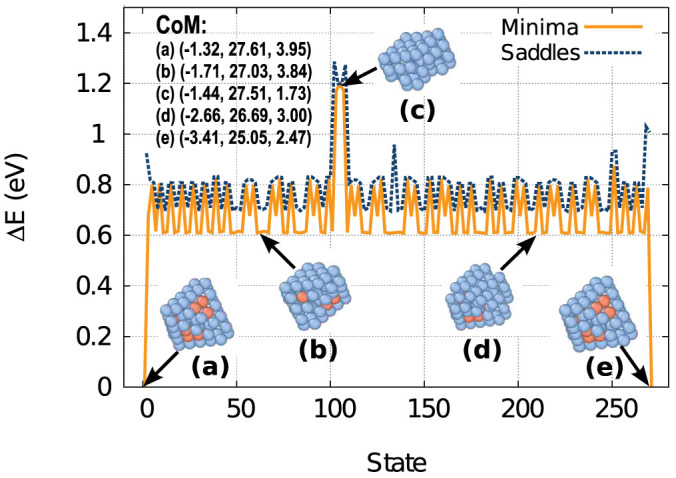
Pathway for the net translation of the CoM of a perfect 10-V SFT as identified from TAD simulations. Only unique states are shown. Both the energies of the minima (solid line) and the saddles (dashed line) relative to the perfect SFT structure at state 0 are indicated. The coordinates of the CoM of each structure in Å are also shown.

Finally, Fig. 6 illustrates a pathway for net migration of the 10-V SFT as identified from the TAD simulations. Both the energies of the visited minima and the saddles connecting those minima are shown, as well as snapshots of the structure of the SFT as a function of the reaction coordinate. First, again, there is a large barrier to deform the perfect SFT (Fig. 6(a)) into a defective one that has higher mobility (Fig. 6(b)). After a number of events that essentially rearrange the atoms comprising the SFT, there is an event in which the cluster collapses to a complex vacancy platelet (Fig. 6(c)), which acts as a gate-way state for the translation of the CoM of the cluster. This event has an even higher barrier, relative to the perfect SFT, of about 1.3 eV. Once it passes through the collapsed state, the SFT first recovers its deformed structure (Fig. 6(d)) and ultimately its perfect configuration (Fig. 6(e)). This shows that the migration of the SFT is a combination of both the rearrangement of the faces due to the surface defects formed when the SFT deforms as well as a massive structural collapse that accompanies the CoM translation of the cluster. The net migration length of the CoM is about 3.6 Å (from Fig. 6(a) to (e)) for this specific case.

It is worth noting that while the total barrier of the SFT migration process is significantly higher than the migration energy of a single vacancy (0.688 eV), their diffusivities as observed in the MD are comparable. We have calculated the entropic contribution to the diffusion rate by obtaining the harmonic prefactor for the 10V-SFT to go from the global minimum to the highest barrier, giving a value of 2.8 10^14^ *s*^−1^, which is about three orders of magnitude larger than for the vacancy. This prefactor is not necessarily representative of the real rate as it is not between states that are connected. However, it gives an indication of the entropic factor for the process and explains why even though the barrier for the 10V-SFT is higher than the one for the vacancy the diffusivities are similar.

In real materials, impurities modify the diffusive properties of most defects, and consequently would influence the behavior of SFTs as compared to what we report in this study. However, the main conclusion of this work will remain unchanged, as the SFTs will have to be considered as mobile entities in higher-level models of deformation and irradiation, which might substantially modify the predicted microstructural evolution. As a matter of fact, a recent *in situ* irradiation study of nano-porous Au^32^ showed the annihilation of a large SFT (~5 nm) at a free surface, which is compatible with the results shown in this work as a defective SFT could migrate to the free surface sink, rather than be completely immobile as has been assumed.

## Conclusions

In view of these results the following main conclusions can be highlighted:

- The large mobility of defected SFTs provides a plausible explanation for the dearth of small tetrahedra in the high temperature regime (above ~500 K) observed experimentally. While Ostwald ripening might be considered as an alternative mechanism for this coarsening, the rate of vacancy emission from the SFTs is smaller than the rate to distort into a mobile structure which makes it less probable (although both mechanisms might be active). Moreover, when a vacancy is ejected from a perfect tetrahedron, the SFT becomes defective, substantially increasing its mobility.
- This study proposes a novel SFT growth mechanism by which small SFTs interact with each other to generate larger ones.
- This work demonstrates that SFT mobility itself might be a critical mass transfer mechanism in the evolution of materials under extreme conditions that must be accounted for in larger scale models to make accurate predictions.
- Even in the simplest of materials (fcc Cu), defect properties can be counter-intuitive, with large vacancy clusters exhibiting mobilities comparable or even in excess of isolated vacancies.

These results shed new light on the existing discrepancies between theoretical models and experimental observations at intermediate and high temperatures, where only large faceted voids are observed as opposed to small and intermediate SFTs. The insight provided by this work explains this observation by demonstrating that SFTs can migrate and coalesce into larger structures that ultimately form the experimentally observed defects.

## Methods

Molecular dynamics (MD) simulations were carried out using the open source code Lammps^33^. The samples were oriented in the [111], 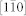 and 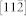 directions with dimensions 3.74 × 3.06 × 3.53 nm for the samples with one SFT that were used to calculate the diffusion coefficients. The sample utilized to study the reaction between a 13V-SFT and a 15V-SFT has dimensions 7.24 × 7.16 × 7.60 nm. The simulations were performed in the NPT ensemble at zero pressure for the varying temperatures specified in the different calculations. Both the total linear momentum and angular momentum were set to zero at each time step. The Mishin embedded atom method (EAM) potential^29^ was used for all the simulations (including TAD). The diffusion coefficient was calculated from 600 ns runs at different temperatures using the Einstein formula (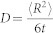) for the coordinates of the center of mass of the atoms with a different from face-centered cubic structure. The analysis was performed after minimizing the sample every 10,000 MD steps.

Temperature Accelerated Dynamics (TAD) involves using a high temperature MD trajectory to explore the current minimum energy basin of the system but extrapolating behavior to lower temperatures to recover the correct state-to-state dynamics (assuming that harmonic transition state theory holds). This procedure can lead to much longer simulation times than possible with direct MD. The simulations were performed using a low temperature of 600 K, high temperatures ranging between 800–1000 K, an uncertainty value of *δ* = 0.01, and an assumed minimum prefactor of 5 10^11^ s^−1^. During the course of the TAD simulation, every event is recorded and characterized with the nudged elastic band method^28^, providing valuable information about the atomic scale mechanisms responsible for the migration of the SFTs.

## Author Contributions

E.M. performed the MD calculations and wrote the first draft of the paper. B.P.U. carried out the TAD calculations. All authors reviewed the manuscript.

## Supplementary Material

Supplementary Information Diffusion 12V-SFT

Supplementary Information SFT reaction

Supplementary Information Diffusion 12V-SFT and 1V
